# *In vitro* maturation of Nelore breed (*Bos taurus indicus*) oocytes using either purified porcine FSH or recombinant human FSH: results from a large-scale *in vitro* embryo production routine

**DOI:** 10.3389/fvets.2025.1596006

**Published:** 2025-07-14

**Authors:** Leticia Prates Martins, Luany Alves Galvão Martinhão, João Gabriel Viana Grazia, Otavio Augusto Costa Faria, Ricardo Alamino Figueiredo, Joao Henrique Moreira Viana

**Affiliations:** ^1^Programa de Pós-Graduação em Biologia Animal, Universidade de Brasília, Brasília, Brazil; ^2^FIVX Apoyar Biotech, Juiz de Fora, Brazil; ^3^Embrapa Recursos Genéticos e Biotecnologia, Brasília, Brazil

**Keywords:** cattle, gonadotropins, *in vitro* maturation, cumulus-oocyte complexes, recombinant hormones

## Abstract

**Introduction:**

This study evaluated whether porcine FSH (pFSH) could be replaced by follitropin-alpha, a recombinant human FSH (rhFSH), during *in vitro* maturation (IVM) of cumulus-oocyte complexes (COC) recovered from Nelore (*Bos taurus indicus*) cattle in a large-scale *in vitro* embryo production (IVP) program.

**Methods:**

We performed three experiments, all using grade I COC (*n* = 10,208) submitted to IVM in TCM199 without FSH (-FSH, negative control) or supplemented with either 0.5 μg/mL pFSH (Folltropin-V) or 0.1 IU rhFSH (Gonal-F). The remaining procedures, media, and culture conditions for *in vitro* embryo production (IVP) were similar. In Exp. 1, COC (*n* = 2,791) were IVM in the absence of FSH (-FSH) or with pFSH or rhFSH, and underwent IVP. Blastocysts were assessed for hatching as fresh or after vitrification and warming. In Exp. 2, COC (*n* = 720) were IVM in groups -FSH, pFSH, or rhFSH, and the expanded blastocysts produced were stained with Hoechst 33342 and propidium iodide for total, trophoblast and ICM cell count. In Exp. 3, we used the same experimental design as in Exp. 1 but in a commercial IVP routine, which included the use of COC (*n* = 6,697) recovered both by OPU and from slaughterhouse, Y-sorted semen, and the transfer of part of the blastocysts produced.

**Results:**

In Exp. 1, blastocyst rates were greater (*p* < 0.05) in groups treated with than without FSH (39.3% and 40.6% vs 34.8% for pFSH, rhFSH and -FSH, respectively; *p* = 0.0244). However, there was no effect of treatment on hatching rates of fresh (74.5%, 79.0% and 70.1% for -FSH, pFSH, and rhFSH, respectively; *p* = 0.2621) or vitrified blastocysts (75.0%, 69.6% and 73.2% for -FSH, pFSH, and rhFSH, respectively; *p* = 0.7623). In Exp. 2, blastocysts from the rhFSH group presented more cells in the trophoblast (110.3±5.6 vs 80.0±5.7 and 878.4±5.1 for rhFSH, -FSH, and pFSH, respectively; P=0.0002), but not in the ICM (*p* = 0.3231), when compared with pFSH and -FSH groups. In Exp. 3, both pFSH and rhFSH increased cleavage (76.0% and 75.1% vs 71.6%, *p* = 0.0035) and blastocyst rates (38.1% and 39.5% vs 34.3%, *p* = 0.0102), but not pregnancy rate (43.8% and 33.3% vs 56.0%, *p* = 0.1080), compared with -FSH.

**Discussion:**

In summary, follitropin-alpha is an alternative to pFSH as a medium supplement for IVM of bovine COC.

## 1 Introduction

During the final steps of folliculogenesis, the FSH, and later, the preovulatory LH peak, trigger cumulus-oocyte complex (COC) maturation within the preovulatory follicle. Oocyte maturation involves a well-coordinated sequence of cytoplasmic and nuclear events that are key for the acquisition of the capacity by the oocyte to undergo fertilization and subsequent embryo development (1). When immature COC are removed from the follicle, they spontaneously resume meiosis, regardless of cytoplasmic maturation (2). Therefore, assisted reproductive technologies (ART) using immature oocytes require an initial step to promote *in vitro* maturation (IVM) of the COC recovered, which is critical for the subsequent *in vitro* embryo production (IVP) outcomes (3).

In most IVM protocols used for IVP in cattle, medium is supplemented with FSH, aiming to mimic the physiological actions of this gonadotropin during follicle maturation *in vivo*. The FSH is known to have a range of effects during COC maturation, including modulation of gene expression patterns (4), protein synthesis (5), glucose uptake and lactate production (6), and extracellular matrix synthesis (7), which leads to cumulus cell expansion and supports acquisition of developmental competence by the oocyte. The FSH formulations currently available for use in livestock are obtained by extraction from porcine hypophysis (pFSH). Although this source of FSH has been used for decades, it has some drawbacks. Due to the impossibility of fully standardizing the raw material used, the biological activity of commercial pFSH varies from batch to batch (8), and presents some degree of contamination with other hormones such as LH and TSH (9). Moreover, there is an intrinsic sanitary risk in the inter-species use of biological extracts, as well as the chance to induce immune recognition (10, 11). Recently, the supply of pFSH has not met the increasing demand for this hormone, resulting in increasing prices or even in a supply shortage in some markets.

In human reproductive medicine, human menopause gonadotropin (hMG) obtained from urine extracts has been replaced by FSH produced using recombinant DNA technologies (12–14). The recombinant human FSH (rhFSH) presents less variation in biological activity and no cross contamination by other hypophyseal hormones (11, 15). Over the past few decades, a number of protocols for ovarian superstimulation in women were developed using rhFSH (16). In non-human species, the use of rhFSH has also been reported, both *in vivo* (17–19) and as a media supplement during IVM of oocytes in pigs (20), cattle (21–23) and sheep (24). However, whether rhFSH is an alternative for pFSH in a commercial IVP routine in livestock still needs to be properly evaluated. Very few studies have directly contrasted pFSH and rhFSH, evaluating a limited number of endpoints and with contradictory results (25, 26). Therefore, up to date the replacement of pFSH by rhFSH for IVP in cattle remains mostly empirical, particularly in breeds other than *Bos taurus taurus*. Moreover, different rhFSH molecules have become commercially available (e.g., follitropin-alpha, −beta, −delta, and -epsilon, corifollitropin), presenting a range of biological activity and pharmacodynamics (11, 27), and rhFSH should not be referred to as a unique molecule. The aim of this study was to evaluate the use of pFSH and follitropin-alpha during IVM of cattle COC, regarding cumulus expansion, blastocyst and hatching rates, embryo morphological quality and cryotolerance, and subsequent pregnancy rates. We hypothesized that pFSH can be replaced by follitropin-alpha without compromising outcomes in a large IVP routine with *Bos taurus indicus* breeds.

## 2 Materials and methods

Unless stated elsewhere, all chemical used were supplied by Sigma-Aldrich (St. Louis, MO, USA).

### 2.1 Experimental design

This study was subdivided into three experiments, all of them using grade I bovine COC recovered *in vivo* by ovum pick-up (OPU) or from slaughterhouse ovaries (mostly Nelore breed, *Bos taurus indicus*). The COC were submitted to *in vitro* maturation (IVM) in Tissue Culture Medium 199 Earle’s salts (TCM199) without FSH (-FSH) or supplemented with either 0.5 μg/mL pFSH (Folltropin-V, Vetoquinol, São Paulo, Brazil) or with 0.1 IU/mL (7.3 ng/mL) rhFSH (follitropin-alpha, Gonal-F, Merck, Darmstadt, Germany). The pFSH dose used was previously tested and adopted as routine by the IVP laboratory enrolled in this study (28). The remaining procedures, media, and culture conditions for *in vitro* fertilization (IVF) and *in vitro* culture (IVC) were similar for all experiments.

Experiment 1 aimed to evaluate the effect of the source of FSH (pFSH or rhFSH) on cumulus expansion, cleavage, blastocyst and hatching rates, and on embryo cryotolerance. COC recovered from slaughterhouse ovaries (*n* = 2,791) were randomly allocated into groups -FSH (negative control), pFSH or rhFSH, and underwent IVP in 10 replicates. Cumulus expansion was evaluated at 22 h of IVM and subjectively scored as absent (−), poor (+) or good (++), as previously described (21). Semen from an Angus sire of known fertility was used for IVF. Cleavage and blastocyst rates were evaluated at days 3, 6, and 7 of IVC. In a subset of the IVP batches (*n* = 945 COC), blastocysts were left in the culture drops and reassessed at day 10 for hatching. In the remaining batches, expanded blastocysts grade I (n = 54, 69 and 71 from -FSH, pFSH and rhFSH, respectively) were IVC after vitrification and warming and evaluated for hatching after 72 h.

Experiment 2 aimed to evaluate the effect of the source of FSH on expanded blastocyst morphological quality, assessed by embryo cell number. COC recovered from slaughterhouse ovaries (*n* = 720) were randomly allocated into groups -FSH, pFSH or rhFSH and underwent IVP in 2 replicates. Expanded blastocysts grade I (*n* = 21, 17 and 15 from -FSH, pFSH and rhFSH, respectively) were submitted to differential staining using Hoechst 33342 and propidium iodide to evaluate the number of cells in the trophoblast, ICM, or total cells, and the ICM: total ratio.

In Experiment 3 we used an experimental design similar to the one described in Exp. 1, but in a commercial IVP routine. COC were recovered from live Nelore (*Bos taurus indicus*) donors by OPU (*n* = 3,521) or from slaughterhouse ovaries (*n* = 3,176), and Y-sorted Angus semen from two sires were used for IVF. In each replicate, COC were randomly allocated into group pFSH (laboratory standard, *n* = 3,536) or to groups -FSH (negative control, *n* = 1,594) or rhFSH (*n* = 1,567), and underwent IVP in 14 replicates. Cleavage and blastocyst rates were evaluated as previously described. A subset of the blastocysts produced (*n* = 1,542) was classified according to developmental stage (early blastocyst, blastocyst, expanded blastocyst, or hatching blastocyst), according to the IETS criteria (29) and nonsurgically transferred to previously synchronized recipients. Pregnancies were diagnosed 23 to 30 days later by B-mode ultrasonography.

All procedures were approved by the Embrapa Ethics in the Use of Animals Committee (Protocol CEUA-001/2022).

### 2.2 Oocyte recovery and *in vitro* embryo production

The COC used were recovered from ovaries collected post-mortem at the Alvorada Slaughterhouse, located at Alta Floresta, MT, Brazil. The ovaries were transported to the laboratory in 0.9% saline at 37°C, where follicles with 3 to 8 mm diameter were aspirated using a 18G needle connected to a 10 mL syringe. The follicular fluid recovered was transferred to 50 mL Falcon tubes kept at 36°C. After decanting, the supernatant was discarded and the pellet was washed three times with 20 mL saline before being transferred to a 100 × 20 mm petri dish (cell culture treated, nonpyrogenic polystyrene, Corning, NY, USA) for identification and selection of grade I COC. In Exp. 3, part of the COC used was recovered by OPU, as previously described (30). The selected COC were washed twice in 60 μL drops of TCM199 and transferred to cryotubes (5 mL polystyrene round-bottom tube, Falcon, Brookings, SD, USA) containing 400 μL of IVM medium and covered with 200 μL mineral oil (Irvine Scientific, Santa Ana, CA, USA). Each tube received circa 30 COC and was labeled according to the experimental group.

The IVM medium consisted of TCM199 supplemented with 22 μg/mL pyruvate and 50 μg/mL amikacin. In all experiments, 10% (v/v) FBS was added. The IVM medium was homogenized, filtered in 0.22 micra membranes and distributed in aliquots, which were then supplemented with 50 μg/mL hCG (Chorulon, MSD, São Paulo, SP, Brazil), 1 μg/mL estradiol, 6.25 μg/mL insulin, 1 μL/mL *β*-mercaptoethanol, and either no FSH, pFSH, or rhFSH, according to the experimental design. The aliquots of media were stored at 5°C until use.

The IVM was performed in incubators at 38.5°C and 5.5% CO_2_ under humidified atmosphere during 22 h. The COC were then transferred to 60 × 15 mm petri dishes (Corning) for evaluation of cumulus expansion, washed with 100 μL IVF medium, and transferred to 50 μL IVF medium drops covered with mineral oil (Irvine Scientific) on 35 × 10 mm petri dishes (Corning). The IVF medium consisted of TALP (Tyrode’s albumin lactate pyruvate) supplemented with 50 μg/mL amikacin, 6 mg/mL fatty acid free BSA, 40 μL/mL of a 2 μM penicillamine solution, 1 μM hipotaurine, 0.25 μM epinephrine and 10 μg/mL heparin. Viable sperm was separated using PureSperm (NidaCon International AB, Mölndal, Sweden), according to manufacturer’s instructions, and circa 1.2 × 10^6^ sperm/mL were added to the IVF drops. Co-incubation was performed during 18 h at 38.5°C and 5.5% CO_2_. After that, the presumptive zygotes were denuded, washed in SOF to remove cumulus cells and sperm, and cultured in 60 μL drops covered with 3.8 mL mineral oil (Irvine Scientific) on 35 × 10 mm petri dishes (Corning), under low oxygen atmosphere (5.5% CO_2_ and 5.5% O_2_, at 38.5°C). Each 10 mL aliquot of SOF was supplemented with 50 μL of 50 μg/mL amikacin, 0.03 g fatty acid free BSA and 300 μL FBS. On days 3 and 6, 70% of the SOF was renewed in each drop.

### 2.3 Vitrification and warming

At day 7 of IVC, a subset of the grade I expanded blastocysts from Experiment 1 was cryopreserved by vitrification, as previously described (31), and stored in liquid nitrogen (N_2_). Briefly, the embryos were washed in two 100 μL drops of IVC media, then placed for 3 min in a 200 μL drop of a vitrification solution with prepared with 7.5% DMSO and 7.5% ethylene glycol, and for 30 s in a 50 μL drop of a vitrification solution with 16.5% DMSO, 16.5% ethylene glycol, and 0.5 M sucrose. The embryos were then individually transferred to an adapted vitrification straw (OPS) and immediately submersed in liquid N_2_. The vitrification straws were stored in racks in a liquid N_2_ container. Embryos were warmed by placing the tip of the vitrification straw in 1 mL of a devitrification solution with 0.25 M sucrose for 1 min. Warmed embryos were then placed in 1 mL of a devitrification solution with 0.15 M sucrose for 5 min, washed in three 60 μL drops of SOF, and submitted to IVC as previously described in section 2.2. Blastocyst re-expansion and hatching rates were evaluated after 72 h.

### 2.4 Differential staining

The differential staining technique used was described elsewhere (32). Briefly, embryos were placed for 30 s in a 500 μL propidium iodine solution (PI 100 μg/mL), prepared with 50 μL PI and 950 μL Triton X 0.3% solution (3 μL of Triton X-100 diluted in 997 μL PBS/PVP), followed by three washes in 500 μL PBS/PVP (50 mL PBS 10 mM plus 0.05 g PVP). Embryos were then transferred to a Hoechst 33342 solution (H33342, 5.0 mg/mL), prepared with 0.2 μL H33342 and 1,000 μL paraformaldehyde 4% (200 μL paraformaldehyde 20% and 800 μL PBS/PVP), for 15 min, followed by three washes in PBS/PVP. All steps were done in four-well dishes (Cultura Nunc, Thermo Scientific Nunc, São Paulo, Brazil). Solutions were kept at 36°C until use. Slowfade (Invitrogen, Waltham, Massachusetts, USA) was used to delimitate a circular area in a glass slide. Embryos (5 per slide) were placed within this circle under a coverslip. The slides were evaluated under fluorescence microscopy (Axiophot 2, Zeiss, Jena, Germany) using a wavelength of 460 nm for the H33342 and 560 nm for the PI. Total, trophoblast and ICM cells were counted under 40X magnification.

### 2.5 Embryo transfer

Embryos from Experiment 3 were transferred to previously synchronized recipients, mostly Nelore breed, raised under pasture with *ad libitum* access to water and mineral supplement. The synchronization protocol consisted of the insertion of an intravaginal progesterone device (1.9 g, CIDR, Zoetis, São Paulo, SP, Brazil) and injection of 2 mg estradiol benzoate IM (Gonadiol, Zoetis) at day −8; removal of the CIDR and injection of 12.5 mg dinoprost (Lutalyse, Zoetis), eCG (Novormon, Zoetis; 200 IU for heifers and 300 IU for cows) at day −2, and 0.6 mg estradiol cypionate (ECP; Zoetis) at day 0 (expected ovulation). At day 7, the ovaries were scanned using an ultrasound device equipped with a linear 5–8 MHz probe (SonoScape E5, Shenzhen, China) to check for the presence and location (right or left) of a corpus luteum. Only recipients with a morphologically functional CL were used. Embryos at blastocyst stage (early blastocysts, blastocysts, or expanded blastocysts) at day 7 of IVC were loaded in 0.25 mL straws in SOF medium supplemented with 5% (v/v) FBS, 0.8 mg/mL HEPES, 3 mg/mL fatty acid free BSA and 0.25 μg/mL amikacin. The straws were sealed and transported to the farms at 37°C in a portable incubator (WTA, Cravinhos, SP, Brazil). Embryos were transferred non-surgically to the uterine horn ipsilateral to the CL.

### 2.6 Data analysis

Aiming to account for effects of replicate (Exp. 1), sire, and source of COC (Exp. 3) within groups, data expressed as percentage (cleavage, blastocyst and hatching rates) were assumed as continuous variables, and were tested for distribution pattern and submitted to ANOVA using the Proc Univariated and the Glimmix procedures of SAS (SAS Studio 3.8, University Edition; SAS Institute Inc., Cary, NC, USA), respectively. In Exp. 1, the model used for IVP outcomes included the effects of group, replicate, and replicate within groups, whereas the model used for hatching rates included the effects of group, cryopreservation, and group x cryopreservation interaction. In Exp. 3 the model included the effects of source of COC (OPU or slaughterhouse), sire, and replicate. In the lack of significant effects within groups, the raw data was analyzed by the Chi-squared method using the Proc Freq of SAS. Quantitative variables (cell counts) were also tested for normality and submitted to ANOVA using the Glimmix procedure. Differences among means were contrasted using the Tukey test. Discrete variables (expansion grade) were analyzed using the Proc Npar procedure of SAS, and differences compared using the Kruskal-Wallis test. A *p*-values of <0.05 was considered as significant. Results are presented as means ± SEM or percentages.

## 3 Results

### 3.1 Experiment 1

**Figure 1:**
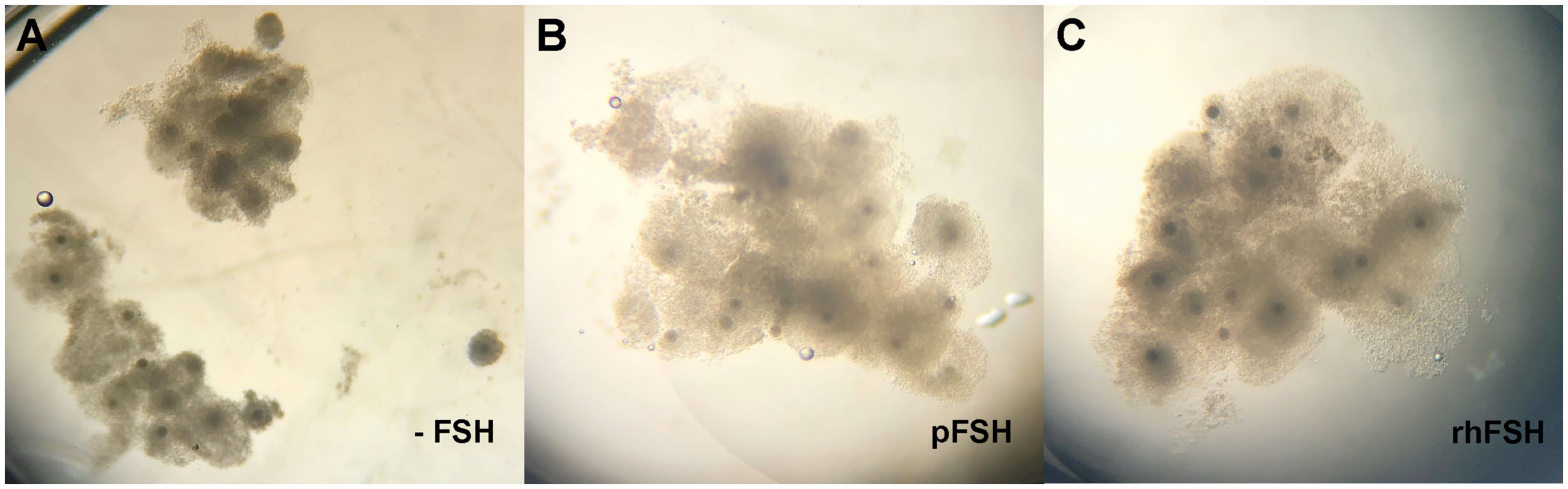
**(A–C)** Expansion of the cumulus cells in bovine COC after 22 h of IVM, according to group. **(A)** -FSH; **(B)** pFSH; **(C)** rhFSH.

All COC from the groups treated with FSH (pFSH and rhFSH) presented good cumulus expansion, which differed (*p* < 0.0001) to the poor expansion observed in the COC that underwent IVM in the absence of FSH (Figure 1).

We observed an effect of replicate (*p* < 0.0001), but not of replicate within group (*p* > 0.05), for blastocyst rates at days 6 and 7. Differences among groups were only observed for blastocyst rate at day 7 (*p* = 0.0258), which was lower for COC matured without FSH (34.8% vs. 39.3 and 40.6% in groups pFSH and rhFSH, respectively). There were no effects of group (*p* = 0.3569), cryopreservation (*p* = 0.2529), or group x cryopreservation interaction (*p* = 0.8079) on hatching rates. The cleavage, blastocyst and hatching rates of fresh or vitrified blastocysts from groups -FSH, pFSH and rhFSH are shown in Table 1.

**Table 1:** IVP outcomes and subsequent hatching rates of fresh or vitrified blastocysts from bovine COC submitted to IVM in the absence (-FSH) or in the presence of porcine (pFSH) or recombinant human FSH (rhFSH).

Endpoint [% (*n*)]	Group			*p*-value
	-FSH	pFSH	rhFSH	
IVP				
Cleavage	77.1% (730/947)	77.2% (658/852)	79.6% (790/992)	*p* = 0.3157
Blastocyst rate (D6)	19.5% (185/947)	21.5% (183/852)	22.3% (221/992)	*p* = 0.3178
Blastocyst rate (D7)	34.8%^b^ (330/947)	39.3%ª (335/852)	40.6%ª (403/992)	*p* = 0.0244
Hatching				
Fresh (D10)	74.5% (82/110)	79.0% (94/119)	70.1% (101/144)	*p* = 0.2621
Vitrified/warmed	75.0% (54/72)	69.6% (48/69)	73.2% (52/71)	*p* = 0.7623

^a,b^Values followed by different superscripts, in the same line, differ (Chi-squared, *p* < 0.05).

### 3.2 Experiment 2

The cell counts after differentially staining expanded blastocysts with Hoescht and propidium iodide are shown in Table 2. Blastocysts obtained from COC matured in the presence of rhFSH presented more trophoblast cells than those from pFSH and -FSH groups (*p* = 0.0002). However, there was no difference in the number of cells in the ICM among groups (*p* = 0.3231). Therefore, embryos from the rhFSH group had more total cells, but less ICM/total ratio, when compared with the negative control (-FSH).

**Table 2:** Number of total, trophoblast and inner cell mass (ICM) cells, as well as the ICM/total ratio, in expanded blastocysts produced from bovine COC submitted to IVM in the absence (-FSH) or in the presence of porcine (pFSH) or recombinant human FSH (rhFSH).

Group	*n*	Total	Trophoblast	ICM	ICM/Total (%)
-FSH	21	132.2^b^ ± 7.3	80.0^b^ ± 5.7	52.2 ± 3.6	39.9^a^ ± 2.5
pFSH	17	134.7^ab^ ± 6.6	87.4^b^ ± 5.1	47.3 ± 3.4	35.2^ab^ ± 2.0
rhFSH	15	153.3^a^ ± 7.7	110.3^a^ ± 5.6	43.0 ± 4.0	27.6^b^ ± 1.9
*p*-value		*p* = 0.0383	*p* = 0.0002	*p* = 0.3231	*p* = 0.0013

^a,b^Values followed by different superscripts, in the same line, differ (*p* < 0.05).

### 3.3 Experiment 3

Sire (35.6% vs. 40.6% for sires A and B, respectively; *p* = 0.0061) and replicate (*p* < 0.0001), but not the source of COC (35.8% vs. 38.8% for slaughterhouse and OPU, respectively; *p* = 0.2726), affected blastocyst rate. However, both sire and replicate effects were evenly distributed within groups -FSH, pFSH and rhFSH. As observed in Exp. 1, both pFSH and rhFSH increased (*p* = 0.0102) blastocyst rates at day 7, compared to the negative control (-FSH). Moreover, we were able to detect also differences in cleavage rates (Table 3) between the groups receiving or not FSH. The use of rhFSH resulted in a smaller proportion of early blastocysts and in a greater proportion of expanded blastocysts, compared with pFSH (*p* < 0.05). Unfortunately, data from a large number of ET were lost due to misidentification of embryo recipients. No difference in pregnancy rates was observed for the remaining transfers (43.8 and 33.3% vs. 56.0% for groups, pFSH, rhFSH, and -FSH respectively; *p* = 0.1080).

**Table 3:** IVP outcomes and subsequent pregnancy rates of blastocysts produced from bovine COC submitted to IVM in the absence (-FSH) or in the presence of porcine (pFSH) or recombinant human FSH (rhFSH), and fertilized with Y-sorted semen.

Endpoint [% (n)]	Group			*p*-value
	-FSH	pFSH	rhFSH	
Cleavage	71.6%^b^ (1,141/1,594)	76.0%ª (2,686/3,536)	75.1%ª (1,177/1,567)	*p* = 0.0035
Blastocyst rate (D7)	34.3%^b^ (546/1,594)	38.1%ª (1,347/3,536)	39.5%ª (611/1,567)	*p* = 0.0102
Early blastocysts (%)	13.5%ª (46/342)	14.6%ª (123/841)	8.6%^b^ (31/359)	*p* = 0.0175
Expanded blast. (%)	73.1%ª (250/342)	66.1%^b^ (556/841)	73.5%ª (264/359)	*p* = 0.0092
Pregnancy rate	56.0% (28/50)	43.8% (28/64)	33.3% (12/36)	*p* = 0.1080

^a,b^Values followed by different superscripts, in the same line, differ (Chi-squared, *p* < 0.05).

## 4 Discussion

In the current study, we evaluated the use of follitropin-alpha, a variant of rhFSH, during IVM of bovine oocytes, and evaluated a range of endpoints of interest. Our results confirm that rhFSH is an alternative to pFSH, whereas the lack of FSH during IVM has a negative impact on COC expansion and on subsequent blastocyst rate, but not on the developmental potential of the embryos produced.

In experiments 1 and 3, we found no differences between pFSH and rhFSH neither in cumulus cell expansion, nor in cleavage, blastocyst, or hatching rates. To our knowledge, this is the first study contrasting pFSH and follitropin-alpha on a large-scale (>10,000 oocytes) and in conditions similar to those of a commercial IVP routine, i.e., using COC recovered both from slaughterhouses and by OPU, and with unsorted or sex-sorted semen. The potential of rhFSH to induce cumulus expansion and meiotic progression (germinal vesicle breakdown, GVBD), in a similar fashion to pFSH, is well known (33, 34). However, a range of concentrations and commercial brands of rhFSH has been empirically used for IVM of bovine oocytes. This could explain the controversial results found in the literature, with greater blastocyst rates being reported for rhFSH, compared with pFSH, by a study (26), but not by others (25, 35). In fact, recombinant FSH preparations have the same primary structure found in the alpha- and beta- subunits of the endogenous FSH, but may differ significantly in glycosylation (36), and thus in biological activity, half-life, and clearance (37). As a result, even biosimilar rhFSH may differ in clinical efficacy (38). In humans, follitropin-alpha was associated with lower estradiol peak concentration, and with greater pregnancy rates after IVF cycles, when compared to follitropin-beta (39). Coherently, the response-curve to rhFSH during IVM also differs among commercial preparations (27).

In the current study we used follitropin-alpha (Gonal-F), an FSH preparation known to promote bovine oocyte maturation in a dose-dependent manner (27). However, concentrations over 1 ng/mL, as the one used in our study (7.3 ng/mL), seem to be effective in promoting cumulus expansion and improving blastocyst rates (25, 26, 34). Taking into account the IVP outcomes, our results confirm the hypothesis that pFSH can be replaced by follitropin-alpha, within the dose used, during IVM of bovine oocytes. Moreover, the supplementation with rhFSH during IVM resulted in blastocysts with more trophoblast cells, as well as in a greater proportion of expanded blastocysts at day 7 of IVC, when compared to pFSH. The trophoblast cells are responsible for the Interferon-Tau (IFNT) production and thus play a key role during maternal recognition of pregnancy (40). Trophectoderm length is positively associated with IFNT gene expression (41). Therefore, the greater number of trophoblast cells in blastocysts from the rhFSH group could, potentially, result in greater pregnancy rates, which is the ultimate goal of the embryo industry (42). However, we could not confirm this hypothesis with our data from embryo transfers, and the biological significance of such differences remain to be elucidated, particularly taking into account that there was no difference in the number of cells in the ICM among groups.

The lack of FSH during IVM consistently resulted in poor cumulus expansion and in lower blastocyst rates across our experiments, pointing out the importance of FSH for COC maturation and acquisition of developmental competence by the oocyte, as reported elsewhere (4, 25, 43). Some studies found no significant positive effect of FSH during IVM on embryo production or quality (6, 44). However, numerical differences in means suggests that such studies may have been underpowered, highlighting the importance of evaluations on a larger scale, as done in the current study. In this regard, it is also noteworthy that blastocyst rates in our negative control group (-FSH) were only circa 10 to 15% lower than those obtained when IVM took place in the presence of FSH. One can speculate whether breed could also affect the response to FSH, as in the current study blastocyst rates were over 34% in all groups. In general, blastocyst rates obtained with beef *Bos taurus indicus* breeds, such as the Nelore, are greater than those from *Bos taurus taurus* (45). Moreover, FSH pre-stimulation before OPU is generally associated with higher embryo rates in *Bos taurus taurus* (46, 47), but not in *Bos taurus indicus* (48).

The relatively small reduction in embryo production, even in the lack of proper cumulus expansion, is also coherent with previous evidence that the presence of cumulus cells, but not cumulus expansion, is required for the acquisition of developmental potential by the bovine oocyte (21, 25). Moreover, it confirms that cumulus expansion has little predictive value for the selection of matured oocytes for IVF, as previously observed for bovine (49) and porcine oocytes (50). Actually, cumulus expansion has an important role during the periovulatory events *in vivo*, as for oocyte retrieval by the fimbriae, which is not required *in vitro*. Similarly, blastocysts produced without FSH presented similar hatching rates, cryotolerance, ICM cell number, and pregnancy rates, to those from pFSH and rhFSH groups. In this regard, we can hypothesize that the lack of FSH during IVM affects the developmental potential of immature oocytes, but not of embryos that were able to develop up to the blastocyst stage. This is coherent with the previous observation that blastocysts have the same chance of developing into pregnancies after transfer, regardless of having originated from IVP batches with high or low blastocyst rates (51).

In summary, supplementation of oocyte maturation media with pFSH or follitropin-alpha results in similar blastocyst rates, and thus the latter is an alternative for the former in a commercial IVP routine. On the other hand, the lack of FSH during IVM results in poor cumulus expansion and in lower blastocyst rate, but has no apparent effect on the developmental potential of the embryos produced.

## Data Availability

The raw data supporting the conclusions of this article will be made available by the authors, without undue reservation.
